# Valerenic acid and *Valeriana officinalis* extracts delay onset of Pentylenetetrazole (PTZ)-Induced seizures in adult *Danio rerio* (Zebrafish)

**DOI:** 10.1186/s12906-015-0731-3

**Published:** 2015-07-14

**Authors:** Bianca A. Torres-Hernández, Lisa M. Del Valle-Mojica, José G. Ortíz

**Affiliations:** Neuropharmacology Laboratory, Pharmacology and Toxicology, Department University of Puerto Rico- Medical Science Campus, PO Box 365067, San Juan, 00936-5067 Puerto Rico

**Keywords:** Herbal-Drug Interaction, Antiepileptic drugs (AEDs), Phenytoin, Clonazepam, Clonic-like seizures, Pentylenetetrazole-induced seizures

## Abstract

**Background:**

Anticonvulsant properties have been attributed to extracts of the herbal medicine *Valeriana officinalis*. Our aims were to examine the anticonvulsant properties of valerenic acid and valerian extracts and to determine whether valerian preparations interact with the activity of other anti-epileptic drugs (phenytoin or clonazepam). To achieve these goals, we validated the adult zebrafish, *Danio rerio,* as an animal model for studying anticonvulsant drugs.

**Methods:**

All drug treatments were administered by immersion in water containing the drug. For assays of anticonvulsant activity, zebrafish were pretreated with: anti-epileptic drugs, valerenic acid, aqueous or ethanolic valerian extracts, or mixtures (phenytoin or clonazepam with valerenic acid or valerian extracts). Seizures were then induced with pentylenetetrazole (PTZ). A behavioral scale was developed for scoring PTZ-induced seizures in adult zebrafish. The seizure latency was evaluated for all pretreatments and control, untreated fish. Valerenic acid and both aqueous and ethanolic extracts of valerian root were also evaluated for their ability to improve survival after pentylenetetrazole-challenge. The assay was validated by comparison with well-studied anticonvulsant drugs (phenytoin, clonazepam, gabapentin and valproate). One-way ANOVA followed by Tukey post-hoc test was performed, using a *p* < 0.05 level of significance. All treatments were compared with the untreated animals and with the other pretreatments.

**Results:**

After exposure to pentylenetetrazole, zebrafish exhibited a series of stereotypical behaviors prior to the appearance of clonic-like movements—convulsions. Both valerenic acid and valerian extracts (aqueous and ethanolic) significantly extended the latency period to the onset of seizure (convulsion) in adult zebrafish. The ethanolic valerian extract was a more potent anticonvulsant than the aqueous extract. Valerenic acid and both valerian extracts interacted synergistically with clonazepam to extended the latency period to the onset of seizure. Phenytoin showed interaction only with the ethanolic valerian extracts.

**Conclusions:**

Valerenic acid and valerian extracts have anticonvulsant properties in adult zebrafish. Valerian extracts markedly enhanced the anticonvulsant effect of both clonazepam and phenytoin, and could contribute to therapy of epileptic patients.

**Electronic supplementary material:**

The online version of this article (doi:10.1186/s12906-015-0731-3) contains supplementary material, which is available to authorized users.

## Background

Epilepsy is a neurological disease with different causes e.g. genetic, structural/metabolic and unknown reasons [1]. In 2012, the Word Health Organization estimate 50 million of people worldwide have epilepsy [2]. Treatment of epileptic patients is difficult because of the inability of current antiepileptic drugs (AEDs) to control some types of seizures in almost 30 % of the patients [2–4]. Other problems associated with current AEDs include low patient compliance [5–7], cognitive problems induced by AEDs [8] and the development of drug resistance [9, 10] among others.

Patients often combine multiple drugs, including natural products, to obtain better seizure control [11], or to treat other health conditions [12, 13] (reviewed in [14]). Interactions of AEDs with other drugs including other anticonvulsants and natural products is a clinical problem [15–17]. Extracts of valerian roots have been used for more than 2000 years to treat a variety of symptoms, such as anxiety and insomnia [18], and for their anticonvulsant properties [19, 20] (reviewed in [21]). It has been documented that patients who self-administer valerian with AEDs may experience increased sedation [21, 22]. Nonetheless, few studies have rigorously examined the anticonvulsant properties of valerian extracts [23–25], and more evidence is required to determine if valerian extracts alter the therapeutic effects of AEDs. The principal objective of the present work was to determine whether valerenic acid or valerian extracts interact with well-studied traditional AEDs phenytoin and clonazepam, to potentiate or reduce their anticonvulsant effect.

*Valeriana officinalis* contains more than 150 chemical constituents identified in the essentials oils [26] and some constituents variation are found from plant harvest [27, 28]. Valerenic acid is considered to be the principal constituent [29, 30]. The aqueous extract contain hydroxylvalerenic acid and acetoxyvalerenic acid in varying proportions [31, 32]. The second objective of the work was to examine the anticonvulsant properties of valerenic acid alone, as well as the aqueous and ethanolic extracts of *Valeriana officinalis.* These studies were performed in adult zebrafish (*Danio rerio*), an animal model used to study clonic-like behaviors [33–36] and for screening drugs [37, 38].

## Methods

### Animals and maintenance

Adult male and female, wild-type short-fin zebrafish (*Danio rerio*), 3–6 months old and weighing 0.25 ± 0.04 g (mean ± SEM) were obtained from Caribe Fisheries Inc. (Lajas, Puerto Rico). Each purchase of 100–200 fish was termed a “batch”. Zebrafish, approximately 1:1 female to male were maintained in an aquarium with an automatic filtration system and covered with blue contact paper to reduce stress. Fish were fed by an automatic feeder two times daily with Wardley Tropical Fish Premium Flakes Food. The animal room was illuminated on a 14/10 h light/dark cycle. Room temperature was maintained at 25 °C ± 1 °C. The animals were observed in quarantine at least one week before use in experimental studies. This study was carried out in strict accordance with the recommendations in the Guide for the Care and Use of Laboratory Animals of the National Institutes of Health. The protocol was approved by the Institutional Animal Care and Use Committee of the University of Puerto Rico, Medical Sciences Campus (Protocol number 3180110). All efforts were made to minimize animal pain and suffering.

### Chemicals

Valerenic acid (VA) was purchased from Chromadex (Irvine, CA.), clonazepam from Roche (Caguas, PR), and phenytoin from Parke-Davis Div of Pfizer Inc. Gabapentin, valproic acid sodium salt and pentylenetetrazole (PTZ) were obtained from Sigma-Aldrich Co. (St. Louis, MO).

The valerenic acid stock solution was prepared by dissolving valerenic acid powder in ethanol (70 %); the stock solution was stored at −20 °C for no more than two months. Dilutions were prepared with aquarium water. The ethanol concentration in valerenic acid solutions (0.40 to 89.5 g/ml) ranged from 0.0059 to 1.4 %, respectively.

Aqueous and ethanolic extracts were prepared from certified organically grown dry powdered roots (Lot 111H-OUP; harvested in 2004) and from fresh valerian roots (Lot 1020P-OUF, harvested in 2008). Both were obtained from Pacific Botanicals (LLC Grants Pass, Oregon).

Extraction methods were modified from Del Valle-Mojica *et al*. [39]. For the ethanolic extract, dry valerian powder (150 mg/ml) was stirred in 70 % ethanol in a covered beaker at room temperature (25 °C) for 1 h. Aqueous extracts were prepared from either fresh root or dry valerian powder (100 mg/ml). Fresh roots were first ground in a blender (Windmere Mod. BD70) at Liquefy mode with Milli-Q ultra-pure water for 2 min. Aqueous preparations were extracted by stirring in a covered beaker for 1 h at room temperature. Particulates were removed by filtering through coarse filter paper (dry powder) or by centrifugation (fresh root) at *6700* g. Extracts were prepared fresh daily for each experiment and were not stored.

### Composition of valerian extracts

Both aqueous and ethanolic valerian extracts of dry valerian powder (Lot 111H-OUP) were prepared and analyzed by Chromadex, Inc. (Irvine, CA) to detect and quantify valerenic acid species (hydroxyvalerenic, acetoxyvalerenic, and valerenic acid) using high-performance liquid chromatography (HPLC). In summary 1250 mg of valerian powder roots were extracted with Milli-Q water or 70 % ethanol in a 25 ml flask and stirred 1 h at room temperature. The extract was then filtered using a 12.5 cm Whatman qualitative #1 filter followed by a 10 min centrifugation at 3700 rpm. The sample was filtered through a 0.45 μm PTFE into an HPLC vial for analysis. Agilent 1100 Series HPLC system was used, with Phenomenex Kinetex C18 30 × 2.1 mm, 2.6 μm column at 50 °C. Elution was done with two mobile phase 0.1 % phosphoric acid and acetonitrile. The injection volume was 0.8 μl with a flow rate of 0.5 ml/min. Detection was done with UV/Vis at 210 nm.

### PTZ experiments

All experiments were performed with randomly selected, untreated male and female zebrafish. Experiments were started between 8:00 am and 4:00 pm. Experiments were performed with 12–16 animals per point, a number which previously provided statistically valid results in adult zebrafish [40].

### PTZ-induced seizures (convulsion) progression scale

The scale was developed with naïve (untreated) zebrafish (2–4 per group, 12–16 fish per concentration). Fish were immersed in a clear plastic tank, (7.5 cm × 4.5 cm × 6.0 cm) containing PTZ (0.1–20 mg/ml) in a final volume of 100 ml aquarium water. Behavior was recorded with a Panasonic SD Video Camera, Model # SDR- S26, for a maximum of 30 min. The video was observed to score the convulsion stages.

### Pretreatments

Prior to the PTZ exposure, naïve zebrafish (3–4 per group) were pretreated by immersion (to minimize stress to animals) for 1 h in a 15 ml chamber (3.0 cm × 5.0 cm × 2.5 cm) containing treatment (valerenic acid, valerian extracts or AEDs) dissolved in aquarium water (Additional file 1). Animals were monitored continuously during the pretreatment period. Because of the toxicity of valerenic acid, pretreatment was for a maximum of 5 min. Possible interactions between AEDs (phenytoin and clonazepam) and valerian extracts were tested with the same protocol, except that the pretreatment tank contained combinations of phenytoin (1 mg/ml) or clonazepam (0.5 μg/ml) with valerian extracts. For valerenic acid combinations with phenytoin or clonazepam, fish were first pretreated for 1 h in water containing the AED, then transferred to a tank containing only valerenic acid (37 μg/ml) for 5 min. Preliminary experiments with a range of concentrations of valerenic acid and valerian extracts were performed to determine the appropriate range for the concentration-response curve. Concentrations higher than 0.1 mg/ml valerenic acid, 2 mg/ml ethanolic valerian extract or 25 mg/ml aqueous valerian extract were not tested due to toxicity (e.g., fish lost posture, lay on its side, had no mobility with exception of the gills (a sign of sedation) or died during the absorption time).

### Latency experiments

Following pretreatment, fish were transferred to a clear plastic tank, (7.5 cm × 4.5 cm × 6.0 cm) containing PTZ (3 mg/ml) in a final volume of 100 ml aquarium water. Behavior was continuously monitored and recorded with a Panasonic SD Video Camera, Model # SDR-S26 for 10 min or until the zebrafish lost posture (maximum of 30 min). The seizure latency period was defined as the time from initial exposure to PTZ until zebrafish reached Stage 7 (defined as a wild jump, clonic-like movement (convulsion), immediately followed by the loss of posture). After the convulsion, animals were removed to the washout tank.

If animals did not show both clonic movements and loss of posture, they were considered to be seizure-free and were not included in the data analysis. The video was observed to score the convulsion stages and to quantify the latency of each fish. Each set of experiments was repeated at least three times using animals from different batches and a minimum of 6 zebrafish per data point. All studies included animals with no pretreatment exposed to PTZ, as daily controls, in addition to animals pretreated with test drugs. The concentrations of AEDs used in drug combination experiments were based on our preliminary concentration-response studies in adult zebrafish.

### Statistical analysis

Analysis of variance (ANOVA) and IC_50_/EC_50_ analyses were calculated with GraphPad Prism software (La Jolla, CA). Latency data are presented as mean ± standard error of the mean (SEM) for individual animals. One-way ANOVA followed by Tukey post-hoc test was performed, using a *p* < 0.05 level of significance. All treatments were compared with the untreated controls and with the other pretreatments.

## Results

### Seizure progression in adult zebrafish

**Fig. 1:**
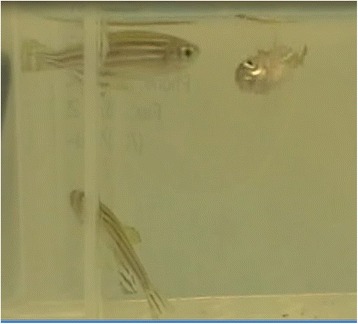
Representative photo of zebrafish after exposure to PTZ 3 mg/ml. Top left , normal posture; top right, laying on its side (Stage 7); bottom, animal with vertical position

Zebrafish exposed to PTZ exhibited a sequence of behaviors before the clonic-like movements (wild jumping) followed by loss of posture that characterized the PTZ-induced seizure. We defined “loss of posture” as the state when the zebrafish lay on its side or had an involuntary vertical position (Fig. 1). The appearance of progressive stages was concentration-dependent (Table 1). In Stage 1 animals usually went to the bottom of the tank either did not move or moved only very close to the bottom. After a few seconds, they began to move very rapidly (Stage 2). In Stage 2, the animals increased their locomotion during 10 min of exposure to PTZ. With PTZ concentrations below 0.5 mg/ml, animals did not progress to other stages of our scale during the 30-min observation period. The complete sequence of behavioral stages 2–7 observed with PTZ concentrations of 0.5 mg/ml and higher included the following: Increased locomotion (Stage 2); hit the tank walls (Stage 3); go to the water surface and then rapidly to the bottom of the tank (Stage 4); faster, occasional shaking movement (Stage 5); and erratic circular or diagonal movement (Stage 6). Stage 7 was characterized as wild jump, clonic-like movement (convulsion), and loss of coordination/posture, staying at the bottom and later recovering posture and motion. This stage could be identified from 0.7 to 3 mg/ml (5–22 mM) PTZ. The progression between stages at higher PTZ concentrations (6–20 mg/ml; 43–144 mM), was very fast, and we could only identify the more obvious stages (1, 2, 6, 7, and 9). Stage 7 was the most clearly reproducible behavior regardless of the pharmacological pretreatment, and for that reason was selected as the end point for quantifying latency. If an animal did not present stage 7, it was considered a seizure-free animal.

**Table 1:** Progression toward clonic-like movement with PTZ in adult zebrafish

Stage	Stage description	PTZ Concentration at which the behavior is first seen
1	Stay or move near the bottom of the tank	0.10 mg/ml (0.72 mM)
2	Rapid movement in all directions,	0.10 mg/ml (0.72 mM)
3	Hit the tank walls	0.50 mg/ml (3.6 mM)
4	Rapidly go to the water surface and then rapidly go to the bottom of the tank	0.70 mg/ml (5.0 mM)
5	Shaking movement	^a^
6	Erratic movements (circular motion, diagonal movement)	0.70 mg/ml (5.0 mM)
7	Wild jumping, Clonic-like movement (convulsion) then loss of coordination/ posture, remaining at the bottom	0.70 mg/ml (5.0 mM)
8	Recover posture/motion	0.70 mg/ml (5.0 mM)
9	Death	1 mg/ml^b^ (7.5 mM)

Zebrafish (*n* = 6–12 per concentration) were exposed to different concentrations of pentylenetetrazole (PTZ) for 30 min. The development of convulsions was analyzed by observation of video tapes. At higher concentrations (6–20 mg/ml) some pre-convulsion stages were not observed

^a^Few naive zebrafish presented this stage, but it was observed in zebrafish challenged with PTZ (3 mg/ml, 21.7 mM) after pretreatment with AEDs or valerians

^b^Some animals survived after 30 min

### The PTZ latency concentration-response curve

**Fig. 2:**
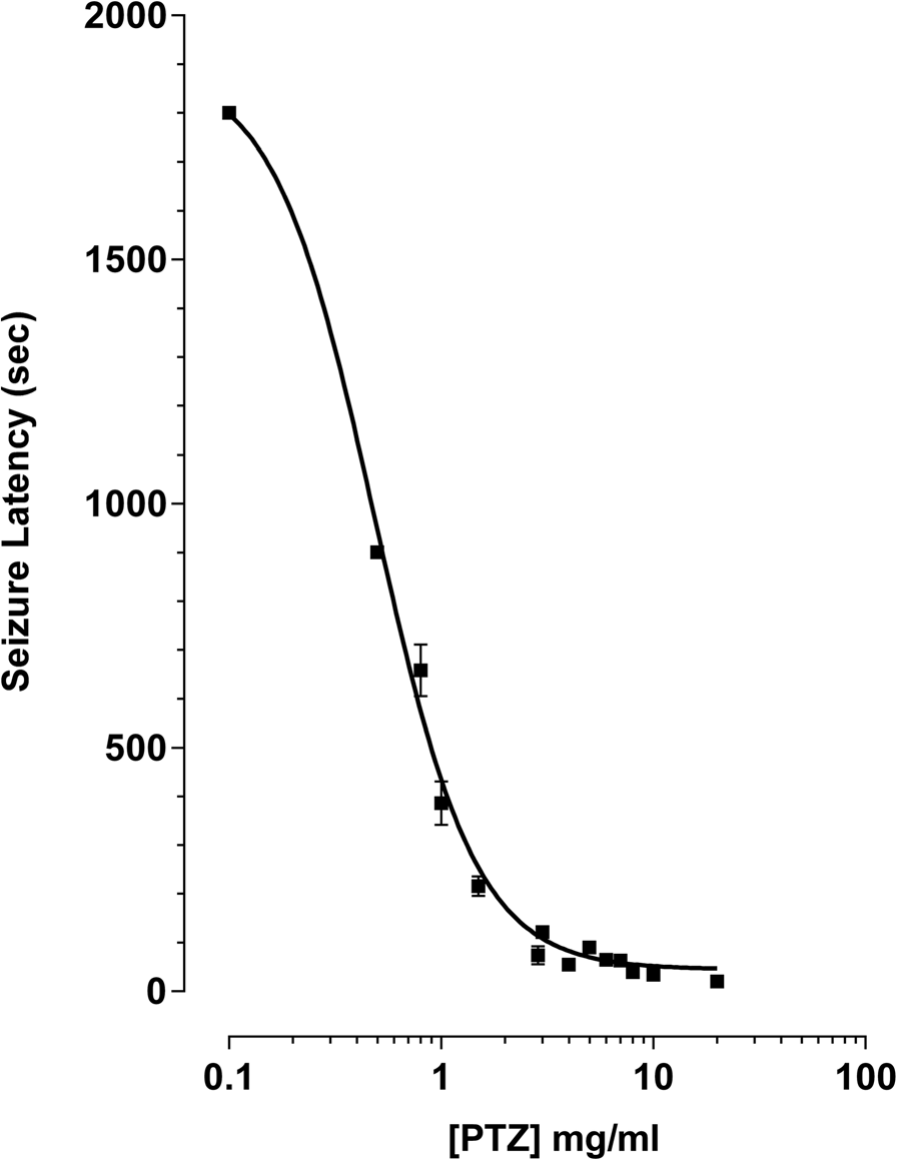
PTZ concentration-response. The latency to Stage 7 PTZ-evoked convulsions was determined in adult zebrafish. Naïve zebrafish (*n* = 6–12) were placed in the convulsion chamber containing PTZ (0.5–20 mg/ml; 0.7–145 mM). The seizure latency period was defined as the time from initial exposure to PTZ until the beginning of Stage 7 (wild jumping immediately followed by loss of posture). The EC_50_ for PTZ-evoked convulsions was 0.49 mg/ml (95 % Confidence Interval, 0.43 to 0.56 mg/ml). Data are mean ± SEM

The seizure latency to the Stage 7, clonic-like movement was determined by exposing zebrafish to a range of PTZ concentrations from 0.7 to 20 mg/ml for 10 min (Fig. 2). Zebrafish did not lose posture during the 10-min exposure to PTZ with concentrations below 0.80 mg/ml. With increasing PTZ concentration (0.70 to 5 mg/ml), the latency to the onset of convulsions decreased in a concentration-dependent fashion. The EC_50_ for PTZ-induced convulsions in adult zebrafish was 0.49 mg/ml (95 % Confidence Interval, 0.43–0.56 mg/ml).

### The effect of traditional AEDs on the latency to convulsion compared with ethanolic valerian extract

**Fig. 3:**
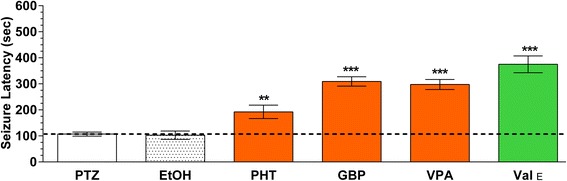
Latency to Stage 7, convulsion-like behavior with antiepileptic drugs (AEDs) and the ethanolic valerian extract. Zebrafish were exposed for 1 h to gabapentin (GBP, 1 mg/ml; 5.8 mM), valproate (VPA, 1 mg/ml; 6 mM), phenytoin (PHT, 2 mg/ml; 7.9 mM), or the ethanolic valerian extract (Val_E,_ 1 mg/ml). All AEDs significantly increased the latency period to Stage 7 PTZ-induced convulsions (PTZ, 3 mg/ml) when compared with untreated zebrafish. Similarly, the ethanolic valerian extract also increased the latency. Ethanol (EtOH) present in the ethanolic valerian extract (0.47 %) had no effect. Data are mean ± SEM of experiments with 9–12 fish per treatment. Inter-group comparisons were by ANOVA. ** *p* < 0.001 or *** *p* < 0.0001 vs PTZ

Naïve (untreated) animals had a seizure latency of 107 ± 8 sec after PTZ challenge (Fig. 3). Traditional AEDs significantly increased the seizure latency after 1 h of pretreatment: gabapentin (1 mg/ml), 309 ± 18 sec and valproate (1 mg/ml), 298 ± 19 sec (*p* < 0.0001); phenytoin (2 mg/ml), 192 ± 26 sec (*p* < 0.001). The ethanolic valerian extract (1 mg/ml) increased the latency to 375 ± 32 sec, significantly greater than all other treatments compare with naïve fish (*p* < 0.0001). Diluted ethanol (0.47 %), the solvent for the ethanolic valerian extract, did not show significant anticonvulsant properties.

### Concentration-response curves of valerenic acid and valerian extracts

**Fig. 4:**
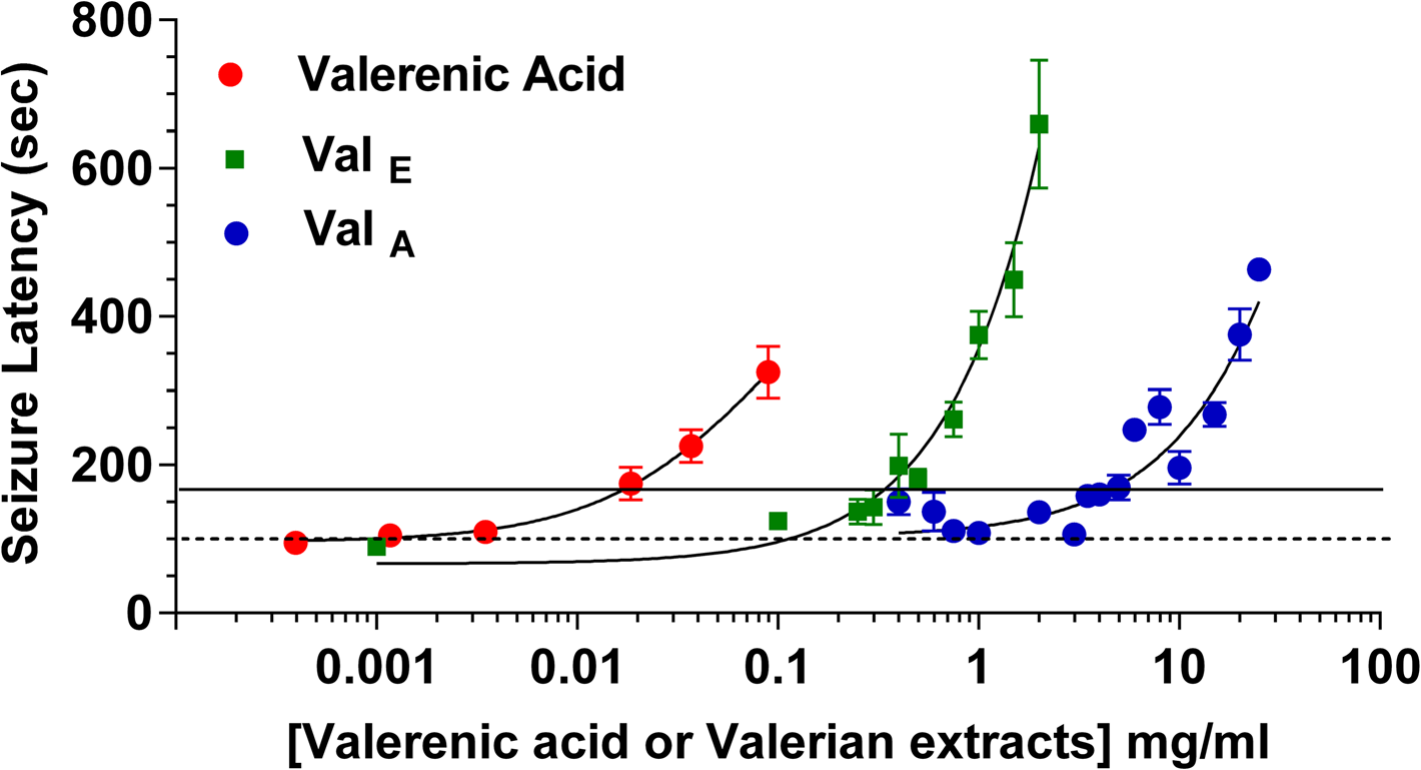
Concentration-response curves of valerenic acid, ethanolic and aqueous valerian extract*.* Zebrafish (*n* = 12–20 fish per concentration) were exposed to valerenic acid (VA), ethanolic (Val_E_) or aqueous (Val_A_) valerian extracts followed by PTZ challenge. The dashed line represents the seizure latency in untreated fish. All values above the solid horizontal line are significantly different from untreated animals, *p* < 0.001 or *p* < 0.0001. (Statistics were omitted for clarity). The threshold concentrations for significant increases in latency were 18.5 μg/ml for valerenic acid, 0.5 mg/ml for the ethanolic valerian extract, and 5 mg/ml for the aqueous valerian extract

The concentration dependency of the ability to delay the latency to convulsion was determined for valerenic acid and the two valerian extracts (Fig. 4). Zebrafish were pretreated for only three minutes with valerenic acid. Concentrations of 18.5 μg/ml and higher (ethanol 0.0059–1.4 %) significantly prolonged the latency to convulsion (*p* < 0.001). Valerenic acid (VA) exhibited toxicity at 89.5 μg/ml and above, with fish exposed for longer than three minutes losing posture in the absorption chamber. The ethanolic valerian extract (Val_E_) was more potent than the aqueous extract (Val_A_), and significantly increased the seizure latency at concentrations 0.50 mg/ml and higher (ethanol 0.23–0.93 %). These ethanol concentrations had no significant anticonvulsant properties (data not shown). The aqueous valerian extract also significantly increased the latency to convulsions at 5 mg/ml or higher (*p* < 0.05). Aqueous extracts prepared from two separate harvests (2004 and 2008) had indistinguishable anticonvulsant effects on latency to PTZ challenge (Additional file 2).

### Interactions of valerenic acid and valerian extracts with AEDs

**Fig. 5:**
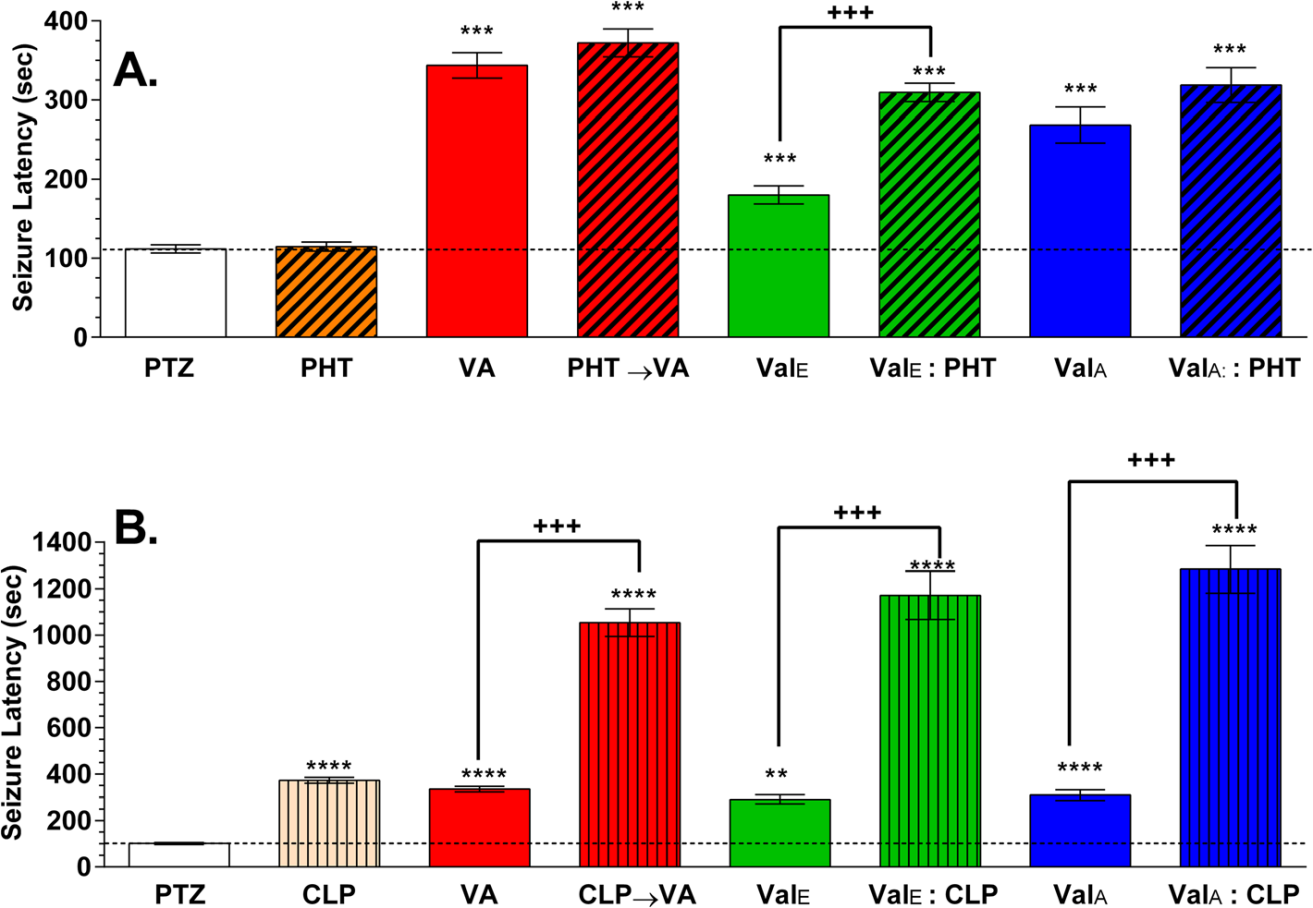
Interaction of valerian extracts or valerenic acid with phenytoin (PHT) or clonazepam (CLP). After 1 h of pretreatment and subsequent exposure to PTZ (3 mg/ml, 21.7 mM). **a** PHT (1 mg/ml, 3.9 mM) had no anticonvulsant effect in zebrafish. However, valerenic acid (VA, 37 μg/ml), ethanolic valerian extracts (Val_E,_ 0.5 mg/ml), and aqueous valerian extracts (Val _A_, 5 mg/ml) and the co-administration of Val_E_ and PHT significantly increased the latency. In contrast, there was no further increase in latency when PHT was co-administered with Val_A_ or before VA. **b** All treatments increased significantly the latency in comparison to untreated fish (PTZ). The combination of clonazepam (5 μg/ml; 0.02 mM) with valerenic acid (37 μg/ml), ethanolic valerian extract (1 mg/ml) or aqueous valerian extract (5 mg/ml) increased the anticonvulsant properties of the individual treatments (*p* < 0.001). The dashed line represents the seizure latency in untreated fish. Data are mean ± SEM from three experiments with 8–21 fish for each treatment, ** *p* < 0.01 or *** *p* < 0.001 or **** *p* < 0.0001 vs untreated; +++ *p* < 0.001 vs treatments alone and PTZ

The possibility that valerenic acid or the extracts (ethanolic or aqueous) could interact either positively or negatively with AEDs was tested by pretreating fish with drug mixtures containing AEDs and extracts prior to PTZ challenge (Fig. 5). Minimally effective concentrations of valerenic acid (37 μg/ml) and valerian extracts (ethanolic extract, 0.5 mg/ml; aqueous extract, 5 mg/ml) were identified from the concentration-response study (unbroken horizontal line in Fig. 4) and were combined with a sub-therapeutic concentration of phenytoin (1 mg/ml). The latency to convulsion in zebrafish treated only with phenytoin was not significantly different from untreated, naïve fish, 112 ± 5 sec. After pretreatment with the ethanolic valerian extract, the latency was 180 ± 12 sec. Pretreatment with the mixture of sub-therapeutic phenytoin and the ethanolic valerian extract (Val_E_:PHT) significantly increased the latency to PTZ challenge to 310 ± 12 sec, around three times that latency of naïve fish, and significantly different from the latency in the ethanolic extract alone (*p* < 0.0001). In contrast, when the aqueous valerian extract was co-administered with phenytoin (Val_A_:PHT), the latency was not significantly different from those pretreated with the aqueous extract alone. Because of the toxicity of valerenic acid in adult zebrafish, fish were first exposed to phenytoin and then to valerenic acid (37 μg/ml) for five minutes (PHT → VA), also failed to show a significant increase in latency compared with fish pretreated with valerenic acid alone (372 ± 18 vs 344 ± 16 sec, respectively).

In contrast with the modest interaction with phenytoin, valerenic acid and valerian extracts strongly potentiated the anticonvulsant effect of clonazepam (5 μg/ml) (Fig. 5b). Zebrafish pretreated with clonazepam and then with valerenic acid (CLP → VA) significantly increased the latency to 1054 ± 59 sec, compared with 335 ± 12 sec in those pretreated with valerenic acid alone. The ethanolic valerian extract (1 mg/ml) combined with clonazepam significantly (Val_E_ : CLP) increased the latency to 1172 ± 104 sec compared with 291 ± 19 sec in fish pretreated with the ethanolic valerian extract alone, or 373 ± 13 sec in fish pretreated with clonazepam alone, and 102 ± 4 sec in untreated naïve animals. The aqueous valerian extract (5 mg/ml) combined with clonazepam (Val_A_ : CLP) also significantly increased the latency to 1284 ± 102 sec, compared with 309 ± 23 sec for those pretreated with the aqueous extract alone. All combinations of clonazepam with valerenic acid or valerian extracts significantly (*p* < 0.0001) potentiated the latency compared to pretreatments with each drug individually. Remarkably, nearly half of the fish (7/16) treated with the mixture of clonazepam and the aqueous valerian extracts did not present clonic-like movement followed by loss of posture during the 30 min observation period. They had normal or fast swimming behavior, and were recorded as seizure-free. All the animals treated with clonazepam plus valerian mixtures survived the half hour exposure to 3 mg/ml PTZ, compared with untreated animals, which only 35 % survived (unpublished data from our lab).

### HPLC analysis of aqueous and ethanolic valerian extracts

Valerenic acid was not detected in the aqueous valerian extract above the reporting limit (0.0051 mg/g of root) and the concentration of acetoxyvalerenic (0.090 mg/g) was lower than in the ethanolic extract (0.796 mg/g). However the amount of hydroxyvalerenic acid was similar in both extracts, 0.193 mg/g vs 0.206 mg/g in the ethanolic and aqueous extracts, respectively.

## Discussion

The zebrafish is an excellent animal model to study behavior and neurological diseases [41–43] including clonic-like behaviors [35, 36], and for screening potential anti-epileptic drugs [37]. We demonstrated that valerenic acid and valerian extracts potentiate the antiepileptic activity of both commercial anticonvulsants phenytoin and clonazepam in adult zebrafish. The progression to PTZ-induced convulsions that we observed in adult zebrafish is very similar to that recently described by Mussulini et al. [33].

To our knowledge, there is no research demonstrating a possible beneficial interaction of valerian extract with antiepileptic drugs. We selected phenytoin and clonazepam, drugs from the first generation AEDs, because they still play an important role in the treatment of clonic convulsions [44, 45]. In the future valproate, gabapentin, and other AEDs should be tested. The positive interaction of ethanolic valerian extracts with phenytoin suggests that adequate concentrations of the ethanolic extract could enhance the effects of sub-therapeutic concentrations of phenytoin. Neither the aqueous valerian extracts nor valerenic acid interact with phenytoin. On the other hand, valerenic acid and both valerian extracts exhibited strong synergism with clonazepam. The mixture of valerians with clonazepam also reduced the risk of mortality in the zebrafish. This suggests that a clonazepam-valerian mixture could be neuroprotective.

In our experiments, the ethanol concentration used as a solvent for the alcoholic valerian extracts and valerenic acid did not have anticonvulsant properties. The final concentration of ethanol in the valerenic acid solution—that did not interact with phenytoin—was the same as the ethanol concentration in the valerian extracts that did interact positively. We do not know if a possible interaction of ethanol with some constituent of the extract are responsible of the observed synergism. Also, we cannot discard the possibility that ethanol could have an effect at higher concentrations in zebrafish or in other animal models. Ethanol reduced the duration of seizure induced by PTZ in mice and enhance the anticonvulsant properties of valproic acid [46].

From the experiments performed, we cannot determine if the interactions between AEDs and extracts were pharmacokinetic, pharmacodynamic or both. Phenytoin in humans is metabolized principally by CYP2C9 and 2C19 [47–49], and clonazepam is metabolized by CYP3A and other P450 cytochrome enzymes [50]. Healthy volunteers have little direct effect on the enzymatic activity of CYP3A4 and CYP2D6 using valerian [51]. Studies *in vitro* with cultured human hepatocytes exposed to common valerian (187.5 μg/ml or 1875 μg/ml) reported induction of CYP2D6 and CYP3A4 but not CYP1A2 [52]. At lower concentrations, weaker induction of CYP2C19 and weaker inhibition of CYP2E1 was reported [53]. Therefore, we think it is likely that pharmacodynamic interactions would better explain our results.

The compounds responsible for the anticonvulsant properties of valerian have not been identified, although many compounds have been identified in valerian extracts, including valepotriates, alkaloids, sesquiterpenes, alcohols, volatile oils, aromatic compounds and other polar and non-polar organic compounds [26, 54]. The medicinal properties of valerian extracts is attributed to valerenic acid, one of the main components in valerian extracts [30]. The HPLC analysis of valerian species present in our extracts found valerenic acid to be present in the ethanolic extract, in addition to hydroxyvalerenic acid and acetoxyvalerenic acid, but valerenic acid was not detected in the aqueous extract although both hydroxyvalerenic acid and acetoxyvalerenic acid were also present. Our data clearly show that pure valerenic acid has strong anticonvulsant properties, but both valerian extracts also have significant anticonvulsant activity. Moreover, the aqueous valerian extract, which did not contain valerenic acid, potentiated the effect of clonazepam more effectively than valerenic acid (Fig. 5b). Thus, valerenic acid is not solely responsible for the anticonvulsant properties of valerian. It is unlikely that hydroxyvalerenic acid is the responsible component, since its concentration is roughly the same in both the aqueous and ethanolic extracts. It is possible that other compounds present in the extracts, or the interaction of various compounds are mainly responsible for the effectiveness of valerian extracts to delay the onset of seizure in fish. Two valerian species that do not contain valerenic acid, *V. edulis* and *V. pavoniis,* also have anticonvulsant properties [55–57].

This work required the development of a PTZ Convulsion Progression Scale for adult zebrafish. The progression toward convulsions was similar to that evoked by injecting adult zebrafish with kainate, an ionotropic glutamate agonist [58]. Our scale is quite similar to a recently published scale for adult zebrafish using a limited range of PTZ concentrations, 5–15 mM [33], whereas we examined concentrations up to 144 mM PTZ. There were several small differences in the behaviors reported. For examples, prior to the erratic movement at Stage 6 we clearly identified two stereotypical behaviors not previously reported, i.e., animals hit the tanks walls (Stage 3) and traveled from the bottom to the surface faster (Stage 4). We also noted that animals could recover posture and motion before death at concentrations of PTZ under 30 mM. We also observed that PTZ was not pro-convulsive at concentrations below 5.8 mM in the first 10 min, but promoted increased locomotor activity, as has been previously reported [59]. Under our conditions, animals did not pass through all PTZ stages after some pretreatments, especially with higher concentrations. Therefore, we decided to use Stage 7 as the end-point to quantify the latency in all experiments.

The only way to assure that the animal have or not a seizure is with an electroencephalography recorded. A limitation in our study was the absence of the activity records and for these reason we named the fish behavior as clonic-like convulsion. If sedation is induced by the treatments, it could delay or masked the clonic-like convulsion. To minimize this possibility we assure that the animal had normal swimming or fast swimming pattern toward the clonic-like movement, whereas sedated animal usually did not move or had slow movement. We observed sedation in those fish treated with higher doses of the valerenic acid, valerian extracts or clonazepam but sedation was not observed at the doses used to determine if the interaction occurs between the treatments.

Our results demonstrated that adult zebrafish can tolerate higher concentrations of both PTZ and AEDs than previously reported for either larval zebrafish [38] or for adult zebrafish [33, 35, 60, 61]. The age-dependent differences in drug response may be attributable to differences in body mass and metabolic capabilities, as has been observed in juvenile and adult rats [62], and humans [63].

## Conclusion

We demonstrate that PTZ challenge in adult zebrafish is a good animal model to study potential antiepileptic drugs. Valerenic acid and *Valeriana officinalis* (both aqueous and ethanolic extracts) increase the latency to PTZ-induced seizure in adult zebrafish in a concentration-dependent manner, with the ethanolic extract being more potent. Ethanolic valerian extracts strongly potentiate the antiepileptic effects of both phenytoin and clonazepam. All valerian preparations and valerenic acid interacted synergistically with a minimally effective concentration of clonazepam to strongly increase the latency to convulsions and improve survival. These results suggest possible new therapeutic alternatives for epileptic patients.

## Additional files

Additional file 1:
**Methodology Representative Schematic.** Show the animals in the absorption chamber and Latency Challenge. Additional file 2:
**Dose–response Curve aqueous extract Valerian harvest 2004 and 2008.** The graph show seizure latency of animals pretreated with selected dose of extract prepared with different harvest roots (2004 and 2008).

## Abbreviations

AEDs: Antiepileptic drugs
CLP: Clonazepam
PHT: Phenytoin
PTZ: Pentylenetetrazole
VA: Valerenic acid
Val_E_: Ethanolic Valerian extract
Val_A_: Aqueous Valerian extract
CYP: Cytochrome P450
