# Supplementary figures and images for: Valerenic acid and Valeriana officinalis extracts delay onset of Pentylenetetrazole (PTZ)-Induced seizures in adult Danio rerio (Zebrafish)
Source: BMC Complement Altern Med. 2015 Jul 14;15:228. doi: 10.1186/s12906-015-0731-3 (PMC4501072; doi:10.1186/s12906-015-0731-3)

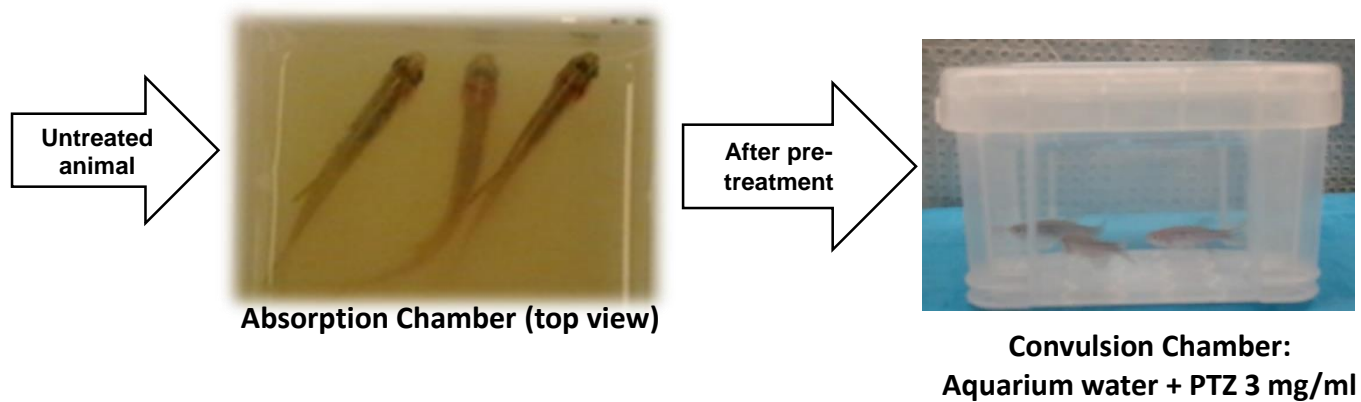

## Additional File 1: Methodology Representative Schematic

Supplement: Additional file 1: — Methodology Representative Schematic. Show the animals in the absorption chamber and Latency Challenge. [file 12906_2015_731_MOESM1_ESM.pdf]

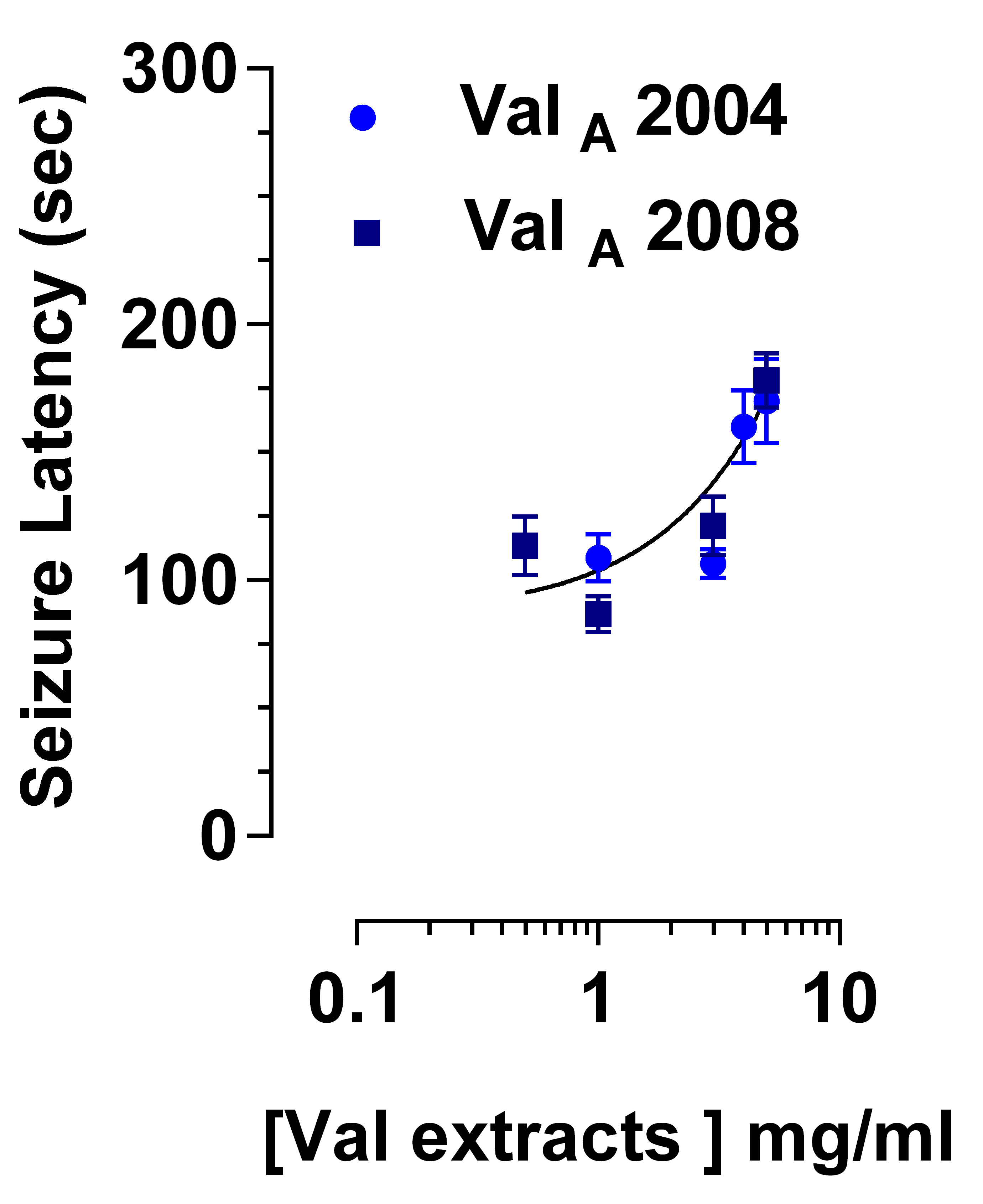

Supplement: Additional file 2: — Dose–response Curve aqueous extract Valerian harvest 2004 and 2008. The graph show seizure latency of animals pretreated with selected dose of extract prepared with different harvest roots (2004 and 2008). [file 12906_2015_731_MOESM2_ESM.tiff]
